# Bacterial Microcompartment-Dependent 1,2-Propanediol Utilization Stimulates Anaerobic Growth of *Listeria monocytogenes* EGDe

**DOI:** 10.3389/fmicb.2019.02660

**Published:** 2019-11-15

**Authors:** Zhe Zeng, Eddy J. Smid, Sjef Boeren, Richard A. Notebaart, Tjakko Abee

**Affiliations:** ^1^Laboratory of Food Microbiology, Wageningen University and Research, Wageningen, Netherlands; ^2^Laboratory of Biochemistry, Wageningen University and Research, Wageningen, Netherlands

**Keywords:** metabolosome, shell protein, 1,2-propanediol utilization cluster, anaerobic growth, pathogen, food safety, virulence

## Abstract

Bacterial microcompartments (BMCs) are proteinaceous organelles that optimize specific metabolic pathways referred to as metabolosomes involving transient production of toxic volatile metabolites such as aldehydes. Previous bioinformatics analysis predicted the presence of BMCs in 23 bacterial phyla including foodborne pathogens and a link with gene clusters for the utilization of host-derived substrates such as 1,2-propanediol utilization, i.e., the Pdu cluster. Although, transcriptional regulation of the Pdu cluster and its role in *Listeria monocytogenes* virulence in animal models have recently been reported, the experimental identification and the physiological role of BMCs in *L. monocytogenes* is still unexplored. Here, we ask whether BMCs could enable utilization of 1,2-propanediol (Pd) in *L. monocytogenes* under anaerobic conditions. Using *L. monocytogenes* EGDe as a model strain, we could demonstrate efficient utilization of Pd with concomitant production of 1-propanol and propionate after 24 h of anaerobic growth, while the utilization was significantly reduced in aerobic conditions. In line with this, expression of genes encoding predicted shell proteins and the signature enzyme propanediol dehydratase is upregulated more than 20-fold in cells anaerobically grown in Pdu-induced versus non-induced control conditions. Additional proteomics analysis confirmed the presence of BMC shell proteins and Pdu enzymes in cells that show active degradation of Pd. Furthermore, using transmission electron microscopy, BMC structures have been detected in these cells linking gene expression, protein composition, and BMCs to activation of the Pdu cluster in anaerobic growth of *L. monocytogenes*. Studies in defined minimal medium with Pd as an energy source showed a significant increase in cell numbers, indicating that Pdu and the predicted generation of ATP in the conversion of propionyl-phosphate to the end product propionate can support anaerobic growth of *L. monocytogenes*. Our findings may suggest a role for BMC-dependent utilization of Pd in *L. monocytogenes* growth, transmission, and interaction with the human host.

## Introduction

Cellular organelles play crucial roles in establishing physical boundaries for biological processes (Yates et al., 2005). Although bacteria lack the classic double membrane-enclosed organelles of eukaryotes, they were found to contain semi-permeable protein compartments known as bacterial microcompartments (BMCs) (Yeates et al., 2008; Kerfeld et al., 2018). BMCs are self-assembling organelles that consist of the encapsulated enzymes and the semi-permeable protein shell playing important roles in metabolic pathways with volatile toxic intermediates (Sutter et al., 2017). A comparative genomic survey previously identified 23 different loci encoding up to 10 functionally distinct BMCs across 23 bacterial phyla (Axen et al., 2014). BMCs may be involved in a range of biological conversions varying from carbon fixation (Rae et al., 2013) to degradation of specific organic compounds such as 1,2-propanediol (Pd) (Bobik et al., 1999; Sampson and Bobik, 2008) and ethanolamine (Garsin, 2010). BMCs are typically about 40–200 nm in diameter and are made of three types of shell proteins: hexamers, pseudohexamers, and pentamers (Kerfeld et al., 2005; Sutter et al., 2017). Hexamers and pseudohexamers are formed by the classical BMC shell proteins containing the Pf00936 domain, while pentamers are formed by the non-classical BMC shell proteins containing the Pf03319 domain (Axen et al., 2014; Sutter et al., 2017). These shell proteins are highly conserved across different bacterial phyla allowing accurate bioinformatics analysis-based prediction of BMCs and associated functions of encapsulated enzymes. Recent evidence has been presented that targeting the encapsulated enzymes to BMCs interior involves the presence of hydrophobic α-helices at the N terminus mediating the interaction with shell proteins (Fan et al., 2012; Aussignargues et al., 2015; Jakobson et al., 2015). Notably, the BMCs that have been characterized and that participate in the heterotrophic metabolism *via* short-chain aldehydes are collectively termed metabolosomes (Aussignargues et al., 2015; Kerfeld and Erbilgin, 2015; Sutter et al., 2017; Kerfeld et al., 2018). These BMCs share a common encapsulated chemistry driven by three core enzymes: aldehyde dehydrogenase, alcohol dehydrogenase, and phosphotransacylase, and their functions have been studied mostly in Gammaproteobacteria including BMCs for the utilization of Pd and ethanolamine in *Salmonella enterica* and *Escherichia coli* and for choline metabolism in *Desulfovibrio desulfuricans* (Bobik et al., 1999; Garsin, 2010; Craciun and Balskus, 2012; Kerfeld et al., 2018). Notably, Pd is a major end product from the anaerobic degradation of rhamnose or fucose by human intestinal microbiota, and it is thought to be an important energy source for Pd utilizing bacteria including pathogens such as *Salmonella* spp. and *Listeria monocytogenes* (Sinha et al., 2012; Jakobson and Tullman-Ercek, 2016). BMC-dependent utilization of Pd in *S. enterica* is encoded on the genome within the Pdu cluster which includes 23 genes: *pocR*, *pduF*, and *pduABCDEGHJKLMNOPQSTUVWX* (Cheng et al., 2011; Sinha et al., 2012). Pd is converted into propionaldehyde by coenzyme B12-dependent diol dehydratase (*pduCDE*), followed by a conversion into 1-propanol and propionate (Cheng et al., 2011). Furthermore, BMC-dependent Pdu in *S. enterica* serovar Typhimurium was found to support its expansion in the murine large intestine (Jakobson and Tullman-Ercek, 2016; Faber et al., 2017). BMC-dependent utilization of Pd by *Listeria* species including the foodborne human pathogen *L. monocytogenes* has gained attention in recent years due to its possible role in pathogenicity (Tang et al., 2015; Schardt et al., 2017). Preliminary bioinformatics analysis indicated that the Pdu cluster in *L. monocytogenes* is mixed with genes encoding enzymes involved in vitamin B12 synthesis and ethanolamine metabolism (Mellin et al., 2013; Chiara et al., 2015). Subsequent studies on transcription regulation identified an important role for a vitamin B12-dependent riboswitch in *L. monocytogenes* (Mellin et al., 2013). Furthermore, a comparative analysis of the virulence characteristics of *L. monocytogenes* EGDe and its PduD knock-out mutant showed that deletion of *pduD* leads to faster clearance in a mouse model of infection (Schardt et al., 2017). This study suggested that Pd metabolism could contribute to competitive fitness of *L. monocytogenes* in the human gastrointestinal (GI) tract and host invasion. Interestingly, transcriptional analysis of *L. monocytogenes* H7858 grown in vacuum-packaged salmon stored at refrigeration temperature revealed activation of genes in the Pdu cluster suggesting that it can support the growth of this pathogen in specific conditions along the food chain thereby contributing to its transmission from soil to host (Tang et al., 2015). However, no experimental evidence has been presented to confirm the synthesis of BMCs in *L. monocytogenes* and to assess their role in Pd metabolism and growth.

Here, we first use a bioinformatics approach to predict and align genes encoding putative BMC shell proteins of the genus *Listeria* and to identify the distribution of the involved Pdu cluster between *Listeria* sensu stricto and *Listeria* sensu lato clades. Using *L. monocytogenes* EGDe as a model, we present evidence for Pd and vitamin B12-induced activation of the genes in the Pdu cluster, Pd metabolism, and production of BMCs using transmission electron microscopy combined with proteomics. In addition, we show that anaerobic growth of *L. monocytogenes* EGDe in defined medium is supported by BMC-dependent utilization of Pd acting as an energy source. Finally, we discuss the possible role of BMC-dependent anaerobic metabolism of Pd in *L. monocytogenes* transmission from soil to host.

## Materials and Methods

### Strains, Culture Conditions, and Growth Measurements

All experiments in this study were carried out with *L. monocytogenes* EGDe and *Listeria grayi* DSM20601. *L. monocytogenes* EGDe and *L. grayi* DSM20601 were grown in Luria Broth (LB) medium and defined medium MWB (Tsai and Hodgson, 2003). LB and MWB were supplemented with Pd and/or vitamin B12 (cobalamin; B12) at final concentrations of 50 mM and 20 nM, respectively (Mellin et al., 2013). Overnight grown cells in LB with or without Pd and vitamin B12 were washed three times in PBS before inoculation into MWB. LB with 50 mM Pd and 20 nM vitamin B12 was defined as Pdu-induced condition, while LB with 50 mM Pd was defined as non-induced control condition. Cultures were incubated at 30°C under aerobic (constantly shaking at 160 rpm) or anaerobic conditions (Anoxomat modified atmosphere, MART; 0% O_2_, 10% CO_2_, 5% H_2_, 85% N_2_) for up to 36 h. OD_600_ measurements were performed every 2 h during the first 12 h of incubation and at 24 and 36 h. Plate counting to quantity Colony Forming Units (CFUs) were performed at 0 and 36 h for experiments performed in defined medium MWB. All growth measurements were performed in three biological replicates with three technical replicates.

### Analysis of 1,2-Propanediol Metabolism Using High Pressure Liquid Chromatography

Samples were taken from the cultures at 0, 12, 24, and 36 h. After centrifugation, the supernatant was collected for the HPLC measurements of Pd, 1-propanol, and propionate. The experiment was performed twice with three technical replicates per experiment. Additionally, the standard curves of Pd, 1-propanol, and propionate were measured in the concentration range of 0.1, 1, 10, 50, and 100 mM. HPLC was performed using an Ultimate 3000 HPLC (Dionex) equipped with an RI-101 refractive index detector (Shodex, Kawasaki, Japan), an autosampler and an ion-exclusion Aminex HPX-87H column (7.8 mm × 300 mm) with a guard column (Bio-Rad, Hercules, CA). As the mobile phase 5 mM H_2_SO_4_ was used at a flow rate of 0.6 ml/min, the column was kept at 40°C. The total run time was 30 min and the injection volume was 10 μl.

### RNA Isolation, cDNA Synthesis, and Quantitative Real-Time PCR

*L. monocytogenes* cultures were grown anaerobically at 30°C in Pdu-induced or non-induced control conditions. Samples for RNA extraction were taken at 8 h of incubation (late exponential phase). RNA isolation was performed with RNeasy Mini Kit (Qiagen). cDNA synthesis was performed with the Superscript III inverse transcriptase (Invitrogen). Supplementary Table S1 lists all primers used in this study. The primers were designed with the Primer3 online software, and their specificity was checked by gel electrophoresis of the PCR products. Primer efficiency was checked by the standard curve method. The efficiencies of all primers ranged from 1.99 to 1.89 (*R*^2^ above 0.99). The Quantitative Real-Time PCR (qRT-PCR) was performed using SYBRgreen PCR MasterMix (Applied Biosystems) in a Bio-Rad CFX96 RT-PCR as described before (Metselaar et al., 2015). Relative expression of the target genes in Pdu-induced versus non-induced control conditions was analyzed with the ΔΔCt method, and expression levels were normalized with the reference gene *rpoB* (Mellin et al., 2013). Samples were evaluated in triplicate and results represent three independent experiments. Statistically significant differences were calculated by using log2 transformed values evaluated by paired *t* test.

### Transmission Electron Microscopy

*L. monocytogenes* cultures were grown anaerobically at 30°C in Pdu-induced or non-induced control conditions. Samples were collected at 12 h of incubation (early stationary phase). About 10 μg dry cells were fixed for 2 h in 2.5% (v/v) glutaraldehyde in 0.1 M sodium cacodylate buffer (pH 7.2). After rinsing in the same buffer, a post-fixation was done in 1% (w/v) OsO_4_ and 2.5% (w/v) K_2_Cr_2_O_7_ for 1 h at room temperature. The samples were dehydrated by ethanol and were then embedded in resin overnight at 70°C. Thin sections (<100 nm) of polymerized resin samples were obtained with microtomes. After staining with 2% (w/v) aqueous uranyl acetate, the samples were analyzed with a Jeol 1400 plus TEM.

### Proteomics

*L. monocytogenes* cultures were anaerobically grown at 30°C in Pdu-induced and in non-induced control conditions. Samples were collected at 12 h of incubation and then washed twice with 100 mM Tris (pH 8). About 10 mg wet weight cells in 100 μl 100 mM Tris was sonicated for 30 s twice to lyse the cells. Samples were prepared according to the filter assisted sample preparation protocol (FASP) (Wiśniewski et al., 2009) with the following steps: reduction with 15 mM dithiothreitol, alkylation with 20 mM acrylamide, and digestion with sequencing grade trypsin overnight. Each prepared peptide sample was analyzed by injecting (18 μl) into a nanoLC-MS/MS (Thermo nLC1000 connected to a LTQ-Orbitrap XL) as described previously (Lu et al., 2011; Wendrich et al., 2017). LCMS data with all MS/MS spectra were analyzed with the MaxQuant quantitative proteomics software package (Cox et al., 2014) as described before (Smaczniak et al., 2012; Wendrich et al., 2017). A protein database with the protein sequences of *L. monocytogenes* EGDe (ID: UP000000817) was downloaded from UniProt. Filtering and further bioinformatics and statistical analysis of the MaxQuant ProteinGroups file were performed with Perseus (Bielow et al., 2015). Reverse hits and contaminants were filtered out. Protein groups were filtered to contain minimally two peptides for protein identification of which at least one is unique and at least one is unmodified. Also, each group (Pdu-induced and non-induced control) required three valid values in at least one of the two experimental groups. The volcano plot was prepared based on the Student’s *t*-test difference of Pdu-induced/non-induced control.

### Bioinformatics Analysis

#### Comparative Genomics of Bacterial Microcompartment Shell Protein Domains and the 1,2-Propanediol Utilization Cluster

The Hidden Markov Models (HMMs) of two BMC shell protein domains listed as Pf00936 and Pf03319 were retrieved from the Pfam database to predict BMC shell proteins in *Listeria* species. The genus *Listeria* is currently comprised of 17 species (Babicki et al., 2016). Shell proteins were predicted across 17 *Listeria* species by a HMM search using the HMMER package and a local protein database of *Listeria* genomes (Mistry et al., 2013). All hits with an *e*-value less than or equal to 1e-05 that correspond to a genomic record from Genbank, RefSeq, EMBL, or DDBJ databases were accepted as BMC shell protein homologs.

To determine which genes from the Pdu cluster are present and which ones are absent in 17 *Listeria* species, we performed BLASTp using the MultiGeneBlast local programme (Medema et al., 2013). In order to obtain the phylogeny of the *Listeria* species, we used a maximum likelihood phylogenetic tree that is based on a concatenated amino acid sequence of 325 single copy genes present in all *Listeria* species (Weller et al., 2015). The heat map visualizing the presence/absence of the Pdu proteins across the tree has been generated with Heatmapper (Babicki et al., 2016) using the similarity values of the protein sequences (expressed in %).

#### Secondary Structure of N Terminal Peptides

The N terminal secondary structures of all Pdu genes were determined by a neural network secondary structure prediction called Jpred 4 (Drozdetskiy et al., 2015). The input to the Jpred 4 online server^1^ was the 40 N terminal amino acids of each protein. Jnetconf: confidence estimation for the prediction with high scores indicating high confidence. Jnetsol25: solvent accessibility, where B means buried and ‘-’ means non-buried at 25% cut-off.

## Results

### Features and Alignment of the *Listeria* spp. 1,2-Propanediol Utilization Cluster

**Figure 1:**
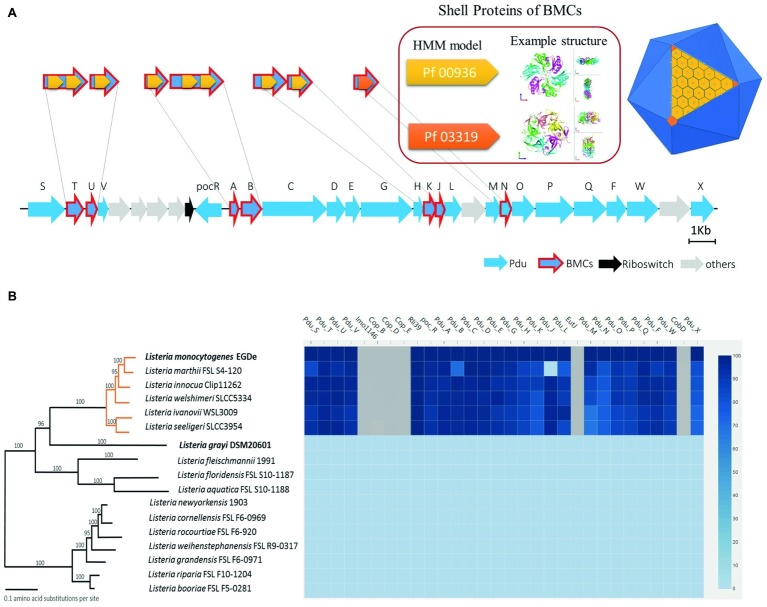
Features, distribution, and alignment of the 1,2-propanediol utilization (Pdu) gene cluster in *Listeria* species. **(A)** Scheme of the Pdu gene cluster with BMC presented as an icosahedron in dark blue with one side showing the assembly principle. The classical BMC shell proteins with the Pf00936 domain form hexamers (presented in light orange) to build the surface blocks of the shell, based on the 3D model described by Kerfeld and Yeates et al. The non-classical BMC shell proteins with the domain Pf03319 form pentamers (presented in dark orange) that constitute the vertex blocks of the shell; PduTUABKJ contains Pf00936, while PduN contains Pf00319. Genes encoding Pdu enzymes are presented in blue; genes encoding BMC shell proteins have an additional red outline; riboswitch genes (Riboswitch) are presented in black and non-Pdu genes (others) presented in gray. See Supplementary Table S3 for additional information of the genes in the Pdu cluster. **(B)** Phylogenetic tree and corresponding similarity heat map of the Pdu cluster in 17 *Listeria* species; orange lines represent *Listeria* sensu stricto, black lines represent *Listeria* sensu lato species. Values on branches represent bootstrap values (>70%) based on 250 bootstrap replicates; Bar, 0.1 amino acid substitutions per site. Protein similarity percentages (%) of the Pdu genes are indicated by blue color intensity according to the scale presented to the right. Species with their names in bold are used in subsequent experiments. Modified from Weller et al. (2015).

Previous structural studies of BMCs revealed that they are icosahedron-shaped and composed of two types of shell proteins present as hexamers or pseudohexamers (domain Pf00936) and pentamers (domain Pf03319) (Sutter et al., 2017; Kerfeld et al., 2018). A whole genome search of Pf00936 and Pf03319 domains revealed that the Pdu cluster in *L. monocytogenes* EGDe contains seven genes encoding for BMC shell proteins, PduTUABKJN (Figure 1A). Next, we analyzed the genomes of 17 *Listeria* species and the phylogenetic tree was modified from Weller et al. (2015) to include *Listeria* sensu stricto and *Listeria* sensu lato (Figure 1B). The genome similarity heat map illustrates the evolutionary dynamics of the Pdu cluster and reveals that except for the loss of *pduJ* in *L. marthii* FSL S4-120, the Pdu cluster is highly conserved in *Listeria* sensu stricto species but absent in *Listeria* sensu lato species, in line with previous studies of *Listeria* sensu stricto and *Listeria* sensu lato species (Chiara et al., 2015; Schardt et al., 2017). Furthermore, the presence of two essential types of BMC shell proteins with Pf00936 and Pf03319 domains in the Pdu cluster of *Listeria* sensu stricto species indicates that all these species including *L. monocytogenes* contain the genetic information to produce BMCs and to metabolize Pd.

### Impact of 1,2-Propanediol on Growth

**Figure 2:**
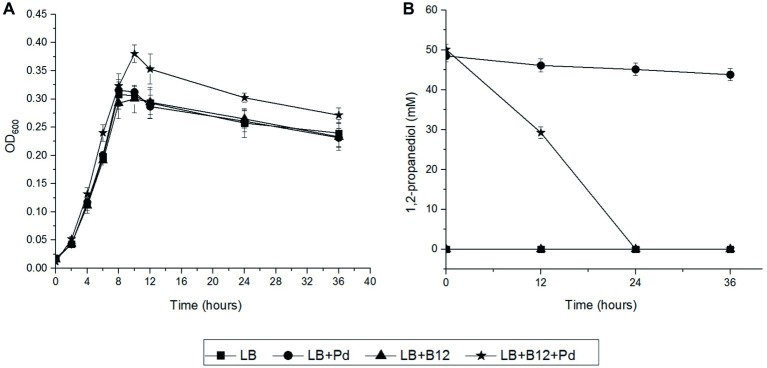
Anaerobic growth and utilization of Pd in *L. monocytogenes* EGDe. **(A)** Impact of Pd and/or vitamin B12 on anaerobic growth of *L. monocytogenes* EGDe in LB medium. Symbols represent different growth conditions; Luria broth without (LB) and with added Pd (LB + Pd), with added vitamin B12 (LB + B12), and with both compounds added (LB + Pd + B12). ANOVA with *post-hoc* Tukey test, LB + Pd + B12 versus LB, LB + Pd, or LB + B12, *p* < 0.001 in 10, 24, and 36 h. Results from three independent experiments with three technical repeats expressed as mean ± s.e.m. **(B)** Utilization of Pd by *L. monocytogenes* EGDe. Symbols used are the same as in Figure 2A. Error bars in **(A,B)** indicate three independent experiments with three technical repeats expressed as mean ± S.E.M.

We first examined the impact of Pd as an additional energy source during anaerobic and aerobic growth of *L. monocytogenes* EGDe in LB medium using Pdu-negative *L. grayi* DSM20601 as a control. The culture medium also includes vitamin B12 as it is required for activation of the Pdu cluster in *L. monocytogenes* (Mellin et al., 2013) and to act as a cofactor (Staib and Fuchs, 2015). In anaerobic conditions, *L. monocytogenes* EGDe grown in Pdu-induced condition, LB medium with Pd and B12, reached significantly higher OD values compared to that obtained in non-induced conditions (Figure 2A). HPLC analysis revealed that *L. monocytogenes* EGDe grown in LB medium with Pd and B12 fully utilized 50 mM Pd after 24 h of anaerobic incubation, whereas no utilization was observed in the other conditions (Figure 2B). Notably, in aerobic shaken EGDe cultures in LB with added Pd and B12, no growth stimulation was observed, which is in line with the HPLC analysis that revealed no utilization of Pd (Supplementary Figure S1). As expected, Pdu-negative *L. grayi* DSM20601 showed no stimulation of growth and no utilization of Pd in both anaerobic and aerobic conditions (Supplementary Figures S2, S3).

Next, we addressed the question whether Pd can act as an energy source in *L. monocytogenes*. To this end, we incubated *L. monocytogenes* EGDe in MWB defined medium with 50 mM Pd and without 20 nM vitamin B12, representing Pdu-induced and non-induced control conditions, respectively. As shown in Supplementary Figure S4, Pdu-induced cells containing BMCs support growth with concomitant production of 1-propanol and propionate, while non-induced control cells without BMCs showed no utilization of Pd and no increased growth. We therefore conclude that activation of the Pdu cluster supports growth of *L. monocytogenes* EGDe with Pd as an energy source.

### Expression Analysis of Selected Genes in the 1,2-Propanediol Utilization Cluster, Pd Metabolism, and Visualization of Bacterial Microcompartments

**Figure 3:**
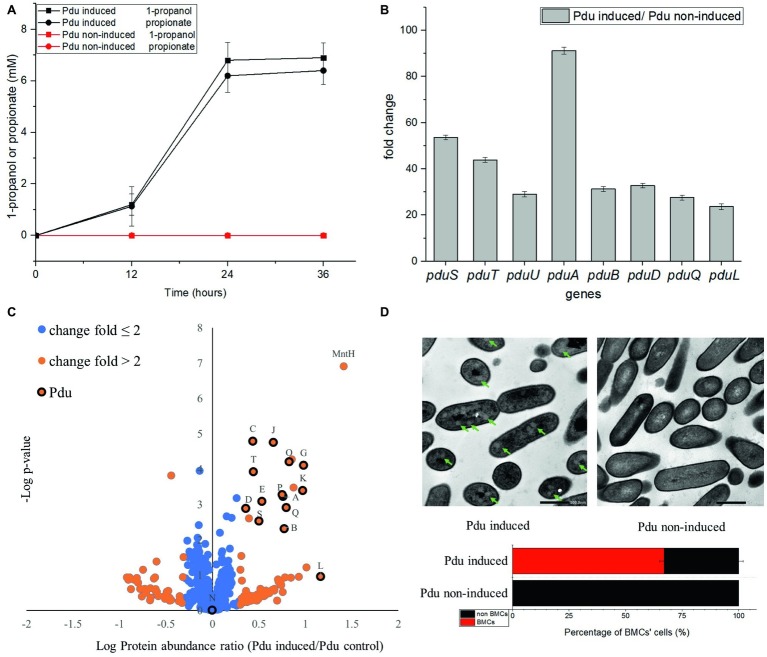
Comparative analysis of product formation **(A)**, gene expression **(B)**, proteomics **(C)**, and presence of BMCs **(D)** in Pdu-induced and non-induced control cells. **(A)** Production of 1-propanol and propionate in *L. monocytogenes* EGDe Pdu-induced (in black) and non-induced control cells (in red). Results from three independent experiments with three technical repeats expressed as mean ± S.E.M. **(B)** Transcription of *pduSTUABDQL* genes. Fold change of selected genes in Pdu-induced cells versus non-induced control cells. Results from two independent experiments with three technical repeats expressed as mean ± S.E.M. **(C)** Proteomic volcano plot of Pdu-induced cells compared to non-induced control cells; fold change ≤ 2 in blue, fold change > 2 in orange, proteins in the Pdu cluster are indicated by their letter code and black circle. MntH, divalent metal cation transporter. **(D)** TEM visualization of BMCs in Pdu-induced (left) and non-induced control cells (right) and percentage of BMC-positive cells. Green arrows point to typical BMC structures, with the black scale bars representing 500 nm.

Utilization of Pd by Pdu-induced cells and non-induced control cells has been analyzed by HPLC and revealed the production of propionate and 1-propanol (Figure 3A). After 24 h anaerobic incubation, Pdu-induced *L. monocytogenes* EGDe cells produced 6.8 ± 0.69 mM 1-propanol and 6.2 ± 0.64 mM propionate, whereas both compounds were not produced by the non-induced control cells (Figure 3A). Production of these two compounds following degradation of Pd was also previously described in *L. innocua* (Xue et al., 2008). Gene expression analysis using qRT-PCR indicated enhanced transcript levels of the selected *L. monocytogenes* EGDe *pduSTUABDQL* genes in Pdu-induced cells compared to non-induced control cells. The *pduS* gene is the starting gene of the Pdu cluster, *pduTUAB* encode four of the seven predicted BMCs shell proteins, *pduD* encodes the subunit of B12-dependent diol dehydratase that converts Pd into propionaldehyde, *pduQ* encodes propanol dehydrogenase that converts propionaldehyde into 1-propanol, and *pduL* encodes phosphotransacylase, that converts propionyl-CoA into propionyl-phosphate (Figure 1A). Figure 3B shows that the selected Pdu genes were upregulated 23 to 91-fold in Pdu-induced cells compared to non-induced control cells. In order to study whether Pdu gene cluster expression is also reflected at the proteome level, we conducted a proteomics analysis. Analysis of the complete list of identified proteins (Supplementary Table S2) and subsequent Student’s *t*-test difference scores of Pdu-induced compared to Pdu non-induced control cells, resulted in a selection of 539 proteins shown in a volcano plot (Figure 3C), with 79 proteins upregulated more than two times in Pdu-induced cells. Among these 79 upregulated proteins, 14 proteins are encoded in the Pdu cluster, i.e., PduSTABCDEGKJLOPQ (Figure 3C), and with PduN present, but not significantly different from non-induced control cells. Clearly, the increased expression of proteins of the Pdu cluster strongly suggests that the catabolism of Pd is correlated to activation of the Pdu cluster including the synthesis of BMC shell proteins. Additional analysis of the set of proteins with more than 2-fold higher protein expression in Pdu-induced cells revealed a range of stress defense proteins including MntA and MntH, components of the divalent metal ion (manganese, Mn) transporter (involved in cytoplasmic transition metal homeostasis and coping with redox stress), LMO2369, general stress protein 13 (cytosolic small ribosomal subunit-associated chaperone linked to a range of stresses including oxidative stress defense), UvrABC system protein C (DNA helicase, linked to DNA damage-induced nucleotide excision repair), ATP-dependent DNA helicase (involved in double-strand break repair *via* homologous recombination), CshB (DEAD-box ATP-dependent RNA helicase linked to oxidative stress response), GbuA, and OpuCA (binding proteins of GbuABC and OpuABC ATP-dependent betaine/carnitine uptake systems involved in osmotic stress defense) (van Schaik and Abee, 2005, Kim et al., 2006, van der Veen et al., 2007, Yu et al., 2009, Markkula et al., 2012, Begg, 2019, Dorey et al., 2019). The significant induction of stress defense and DNA damage repair proteins in Pdu-induced *L. monocytogenes* cells has not been studied and reported before, but is in line with the previously reported DNA and cellular damage of *S. enterica* cells exposed to Pd (Sampson and Bobik, 2008). Creation of BMCs with key metabolic turnover steps of Pdu encapsulated, thereby creating a protection against the toxic intermediate propionaldehyde, is proposed to occur in a stepwise manner (Jakobson and Tullman-Ercek, 2016; Kerfeld et al., 2018). Our observation may suggest that BMC assembly co-occurs with activation of stress defense proteins, possibly to respond to toxic intermediates and redox stress before BMC production is completed.

To further confirm the presence of BMCs in Pdu-induced cells, we used TEM and compared thin sections of both Pdu-induced and non-induced control cells (Figure 3D). Pdu-induced cells clearly contain BMC-like structures with an approximate diameter of 60–80 nm, which are not present in non-induced control cells. Notably, the identified structures strongly resemble TEM pictures of BMCs in *S. enterica* and *E. coli* (Cheng et al., 2011; Dadswell et al., 2019). Taken the metabolic, transcriptomic, proteomic, and TEM data together, we conclude that the cytosolic BMC-like structures in *L. monocytogenes* EGDe are involved in Pd utilization under anaerobic conditions. From the analysis of 400 cells in Pdu-induced samples, 268 cells (67%) contained one or more BMCs, whereas no or partial BMC structures could be observed in the other 33% of cells. This observation highlights the need for further studies at subpopulation level to address the question to what extend activation of stress defense and DNA damage repair are linked to initiation and progression of Pdu BMC assembly and function.

### Prediction of N Terminal Encapsulation Peptides and the Model Depicting Bacterial Microcompartment-Dependent Conversions in *Listeria monocytogenes* 1,2-Propanediol Utilization

**Figure 4:**
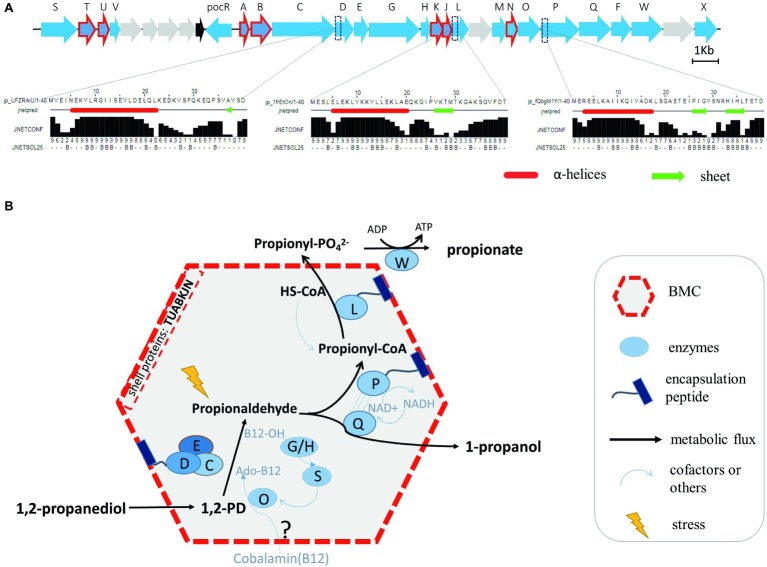
Prediction of N terminal encapsulation peptides **(A)** and the corresponding model of BMC-dependent metabolism of Pd catabolism in *Listeria* sensu stricto **(B)**. **(A)** Pdu enzymes and corresponding reactions, with the predicted N-terminal encapsulation peptides of PduD, PduP, and PduL indicated, with alpha helices marked as red tubes and sheets as green arrows. Jnetconf: confidence estimation for the prediction with high scores indicating high confidence. Jnetsol25: solvent accessibility, where B means buried and ‘-’ means non-buried at 25% cut-off. **(B)** Model of the proposed BMC-dependent Pd metabolism in *Listeria* sensu stricto. PduCDE, B12-dependent diol dehydratase; PduP, CoA-dependent propionaldehyde dehydrogenase; PduGH, diol dehydratase reactivase; PduO, corrinoid adenosyltransferase; PduS, cobalamin reductase; PduL, phosphate propanoyltransferase; PduW, propionate kinase; PduQ, propanol dehydrogenase.

In order to provide further evidence for the putative location of Pd enzyme-mediated conversions in *L. monocytogenes* Pdu-associated BMCs, a comparative analysis of the domains of all the proteins in the Pdu cluster has been performed, with a special focus on domains mimicking N terminal encapsulation peptides (EPs). We indeed identified a specific hydrophobic α-helix in the N terminus of the encapsulated enzyme PduP, similar to that of the previously described encapsulated protein PduP in *S. enterica* (Fan et al., 2012). Based on this knowledge, we predicted the secondary structure of the 40 amino acids sequence in the N terminal domains of all *L. monocytogenes* EGDe Pdu proteins. In addition to PduP, PduD and PduL were also found to contain the hydrophobic N-terminal α-helical EP domain (Figure 4A). Interestingly, PduD has been previously confirmed as an encapsulated enzyme in *S. enterica* BMCs (Fan et al., 2012; Jakobson et al., 2015). The proposed encapsulation of PduL that converts propionyl-CoA into propionyl-phosphate would support efficient recycling of CoA inside the BMC. Based on our analysis, we propose that the PduDPL enzymes are encapsulated in *L. monocytogenes* EGDe Pdu-associated BMCs (Figure 4B, and section “Discussion”).

## Discussion

The proposed model for BMC-dependent Pdu metabolism in *L. monocytogenes* combines bioinformatics analysis, metabolic phenotyping, transcriptional analysis, proteomics, TEM visualization, and experimental verification in defined medium.

As illustrated in Figure 4B, the catabolism of Pd starts with the conversion of Pd to propionaldehyde by vitamin B12-dependent diol dehydratase PduCDE (Sampson and Bobik, 2008; Xue et al., 2008; Sinha et al., 2012). The toxic propionaldehyde is then converted to propionate by the enzyme CoA-dependent propionaldehyde dehydrogenase PduP, followed by action of phosphate propanoyltransferase PduL, and propionate kinase PduW located in the cytoplasm, resulting in the end product propionate and the production of ATP. The other end product is produced following conversion of propionaldehyde by propanol dehydrogenase PduQ into 1-propanol. PduQ is thought to be inside BMCs because of the potential association with PduP (Cheng et al., 2012). The diol dehydratase reactivase PduGH, corrinoid adenosyltransferase PduO, and cobalamin reductase PduS are linked to the supply and recycling of vitamin B12. Predicted shell proteins PduTUABKJ and PduN form the hexamer and pentamer building units of the icosahedron-shaped BMCs for Pdu catabolism shielding the cell constituents against toxicity of the volatile intermediate propionaldehyde and supporting efficient recycling of the CoA cofactor pool. Based on our Pdu metabolism studies, gene expression analysis and proteomics data, and in line with their role in Pdu enzyme activity, we locate the vitamin B12 recycling proteins PduGHOS inside the *L. monocytogenes* BMC. It should be noted that the exact mechanisms underlying the entry of vitamin B12 and the encapsulation of vitamin B12 recycling proteins PduGHOS remain to be elucidated (Kerfeld et al., 2018).

According to the alignment of Pdu clusters in 17 *Listeria* species and in line with previous genome comparisons (Chiara et al., 2015; Schardt et al., 2017), we propose that BMC-dependent Pdu clusters, except for the loss of *pduJ* in *Listeria marthii* FSL S4-120, are present in *Listeria* sensu stricto but absent in *Listeria* sensu lato. Considering the highly conserved Pdu cluster in *Listeria* sensu stricto and the reported degradation of Pd in another *Listeria* sensu stricto species, *L. innocua* (Xue et al., 2008), it is conceivable that the presented BMC-dependent Pdu model also fits Pd metabolism in other *Listeria* sensu stricto species.

Since Pd can be present in processed foods and in the human gut, it is assumed that Pdu BMCs belong to a subset of BMCs that contribute to the fitness and virulence of some pathogenic bacteria including *S. enterica* and *L. monocytogenes* (Staib and Fuchs, 2015; Jakobson and Tullman-Ercek, 2016; Faber et al., 2017; Schardt et al., 2017). The possible contribution of Pdu BMCs to the pathogenicity of *L. monocytogenes* is however still poorly understood, but a recent study using *L. monocytogenes* EGDe and its PduD knock-out mutant showed that deletion of *pduD* leads to faster clearance in a mouse model of infection (Schardt et al., 2017). Notably, our studies demonstrate that Pd utilization indeed stimulates the anaerobic growth of *L. monocytogenes.* Furthermore, studies in defined minimal medium with Pd as an energy source show that the predicted generation of ATP in the conversion of propionyl-phosphate to the end product propionate can support anaerobic growth of *L. monocytogenes*. This suggests that BMC-dependent Pdu contributes to competitive fitness of *L. monocytogenes* in the human GI tract and host invasion. Whether BMC-dependent Pdu contributes to growth of this pathogen in specific conditions along the food chain, as suggested by Tang et al. based on the activation of Pdu cluster genes in *L. monocytogenes* H7858 grown in vacuum-packaged salmon stored at refrigeration temperature (Tang et al., 2015), remains to be established.

Strikingly, the impact of Pdu activation and the timing of BMC assembly on cell physiology and fitness has gained very little attention, although optimization of the process is required to enable enhanced substrate flux and efficient toxicity mitigation (Jakobson and Tullman-Ercek, 2016). Indeed, our proteomics data reveal significant activation of stress defense proteins, including general stress sigma factor Sigma B-controlled genes *gbuA* and *opuC* encoding transporter binding proteins involved in the uptake of compatible solutes (betaine/carnitine) that have a role in cell turgor homeostasis, but also in maintenance of protein/enzyme and membrane functionality (Wemekamp-Kamphuis et al., 2002; van der Veen et al., 2007; Dorey et al., 2019; Marinho et al., 2019), and *uvrAB*, part of the *L. monocytogenes* SOS response, contributing to protection and/or DNA damage repair in the cells (van der Veen et al., 2007). Notably, Liu et al. (2019) identified new Sigma B-dependent functions in *L. monocytogenes* including the regulation of genes involved in Pdu. Combined with our observations, and the previous reported role for a vitamin B12-dependent riboswitch (Mellin et al., 2013), this could point to an integration of signals and responses enabling adequate and timely assembly of *L. monocytogenes* Pdu BMCs. Obviously, further work is needed to understand BMC function at population and systems-level, especially considering the observed population heterogeneity in our studies, with an approximate 67 and 33% of BMC positive versus BMC-negative cells. Such future studies should address to what level the activation of stress defense and DNA damage repair are linked to Pdu BMC assembly and function, and how this affects the role of Pdu BMCs in *L. monocytogenes* transmission and interaction with the human host.

## Data Availability Statement

The raw data supporting the conclusions of this manuscript will be made available by the authors, without undue reservation, to any qualified researcher.

## Author Contributions

ZZ, RN, and TA designed the experiments. ZZ performed the experiments. SB performed proteomics and analyzed data. ZZ, RN, and TA analyzed data. ZZ, RN, and TA wrote the manuscript. SB and ES made improvements.

### Conflict of Interest

The authors declare that the research was conducted in the absence of any commercial or financial relationships that could be construed as a potential conflict of interest.

## Footnotes

^1^http://www.compbio.dundee.ac.uk/jpred4/
