# Supplementary material for: Bacterial Microcompartment-Dependent 1,2-Propanediol Utilization Stimulates Anaerobic Growth of Listeria monocytogenes EGDe
Source: Front Microbiol. 2019 Nov 15;10:2660. doi: 10.3389/fmicb.2019.02660 (PMC6873790; doi:10.3389/fmicb.2019.02660)
Supplement: Supplementary file 2 [file Data_Sheet_1.docx]

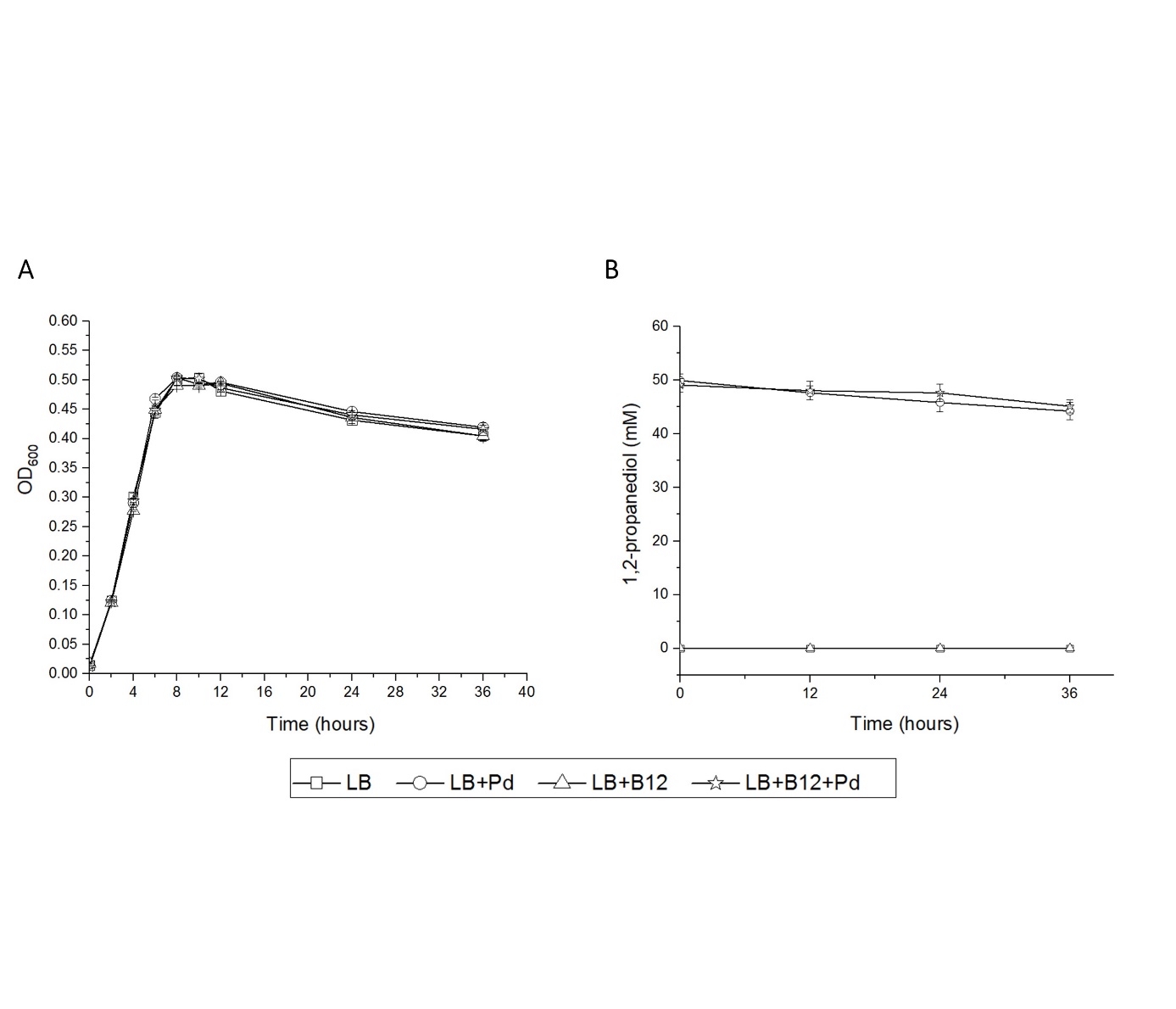


**SI Figure 1. 1,2-propanediol is not utilized and does not support growth of *L. monocytogenes* EGDe in aerobic conditions**

**A)** Impact of Pd and/or B12 on aerobic growth of *L. monocytogenes* EGDe in LB medium. Symbols represent different growth conditions; Luria broth without (LB) and with added 1,2-propanediol (LB+Pd), with added vitamin B12 (LB+B12), and with both compounds added (LB+Pd+B12). ANOVA with post-hoc Tukey test, no significant differences, p>0.05. Results from three independent experiments with three technical repeats expressed as mean ± s.e.m.

**B)** Utilization of 1,2-propanediol by *L. monocytogenes* EGDe during aerobic growth. Results from three independent experiments with three technical repeats are expressed as mean ± s.e.m.


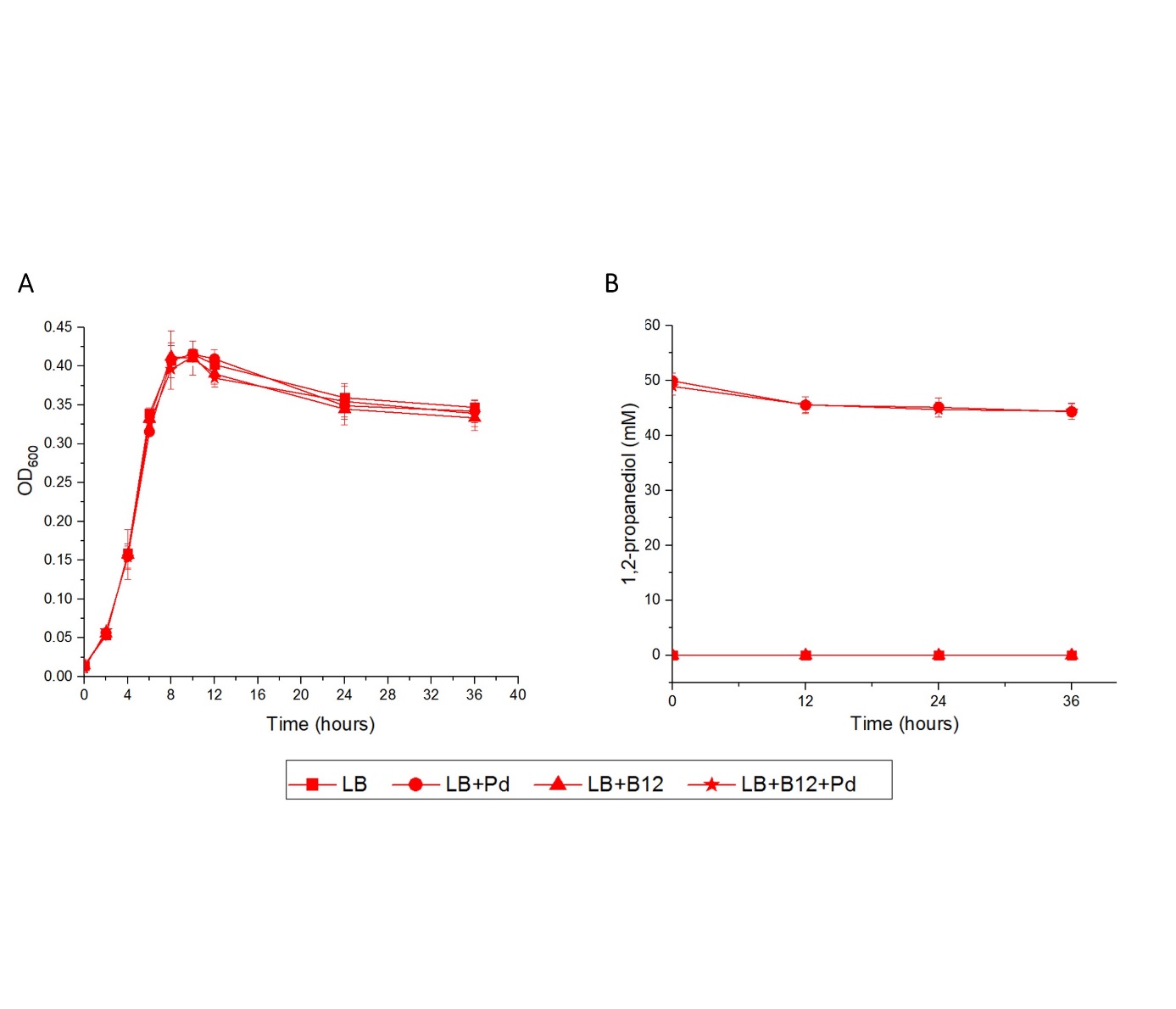


**SI Figure 2. 1,2-propanediol is not utilized and does not support growth of Pdu-negative *L. grayi* DSM20601 in anaerobic conditions**

**A)** Impact of Pd and/or B12 on anaerobic growth of *L. grayi* DSM20601 in LB medium. Symbols represent different growth conditions; Luria broth without (LB) and with added 1,2-propanediol (LB+Pd), with added vitamin B12 (LB+B12), and with both compounds added (LB+Pd+B12). ANOVA with post-hoc Tukey test, no significant differences, p>0.05. Results from three independent experiments with three technical repeats expressed as mean ± s.e.m.

**B)** Utilization of 1,2-propanediol by *L. grayi* DSM20601 during anaerobic growth. Results from three independent experiments with three technical repeats are expressed as mean ± s.e.m.


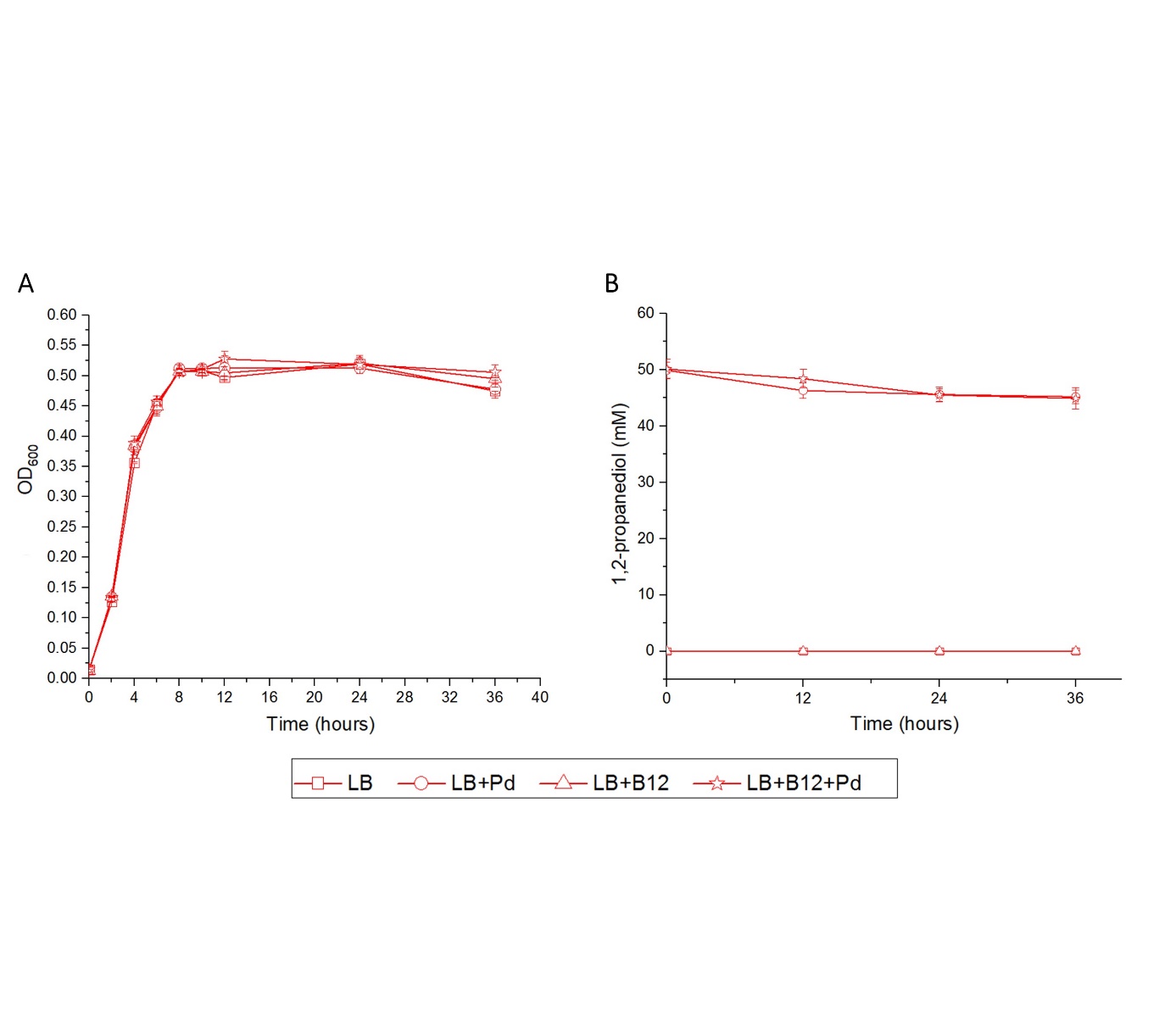


**SI Figure 3. 1,2-propanediol is not utilized and does not support growth of Pdu-negative *L. grayi* DSM20601 in aerobic conditions**

**A)** Impact of Pd and/or B12 on aerobic growth of *L. grayi* DSM20601 in LB medium. Symbols represent different growth conditions; Luria broth without (LB) and with added 1,2-propanediol (LB+Pd), with added vitamin B12 (LB+B12), and with both compounds added (LB+Pd+B12). ANOVA with post-hoc Tukey test, no significant differences, p>0.05. Results from three independent experiments with three technical repeats expressed as mean ± s.e.m.

**B)** Utilisation of 1,2-propanediol by *L. grayi* DSM20601 during aerobic growth. Results from three independent experiments with three technical repeats are expressed as mean ± s.e.m.


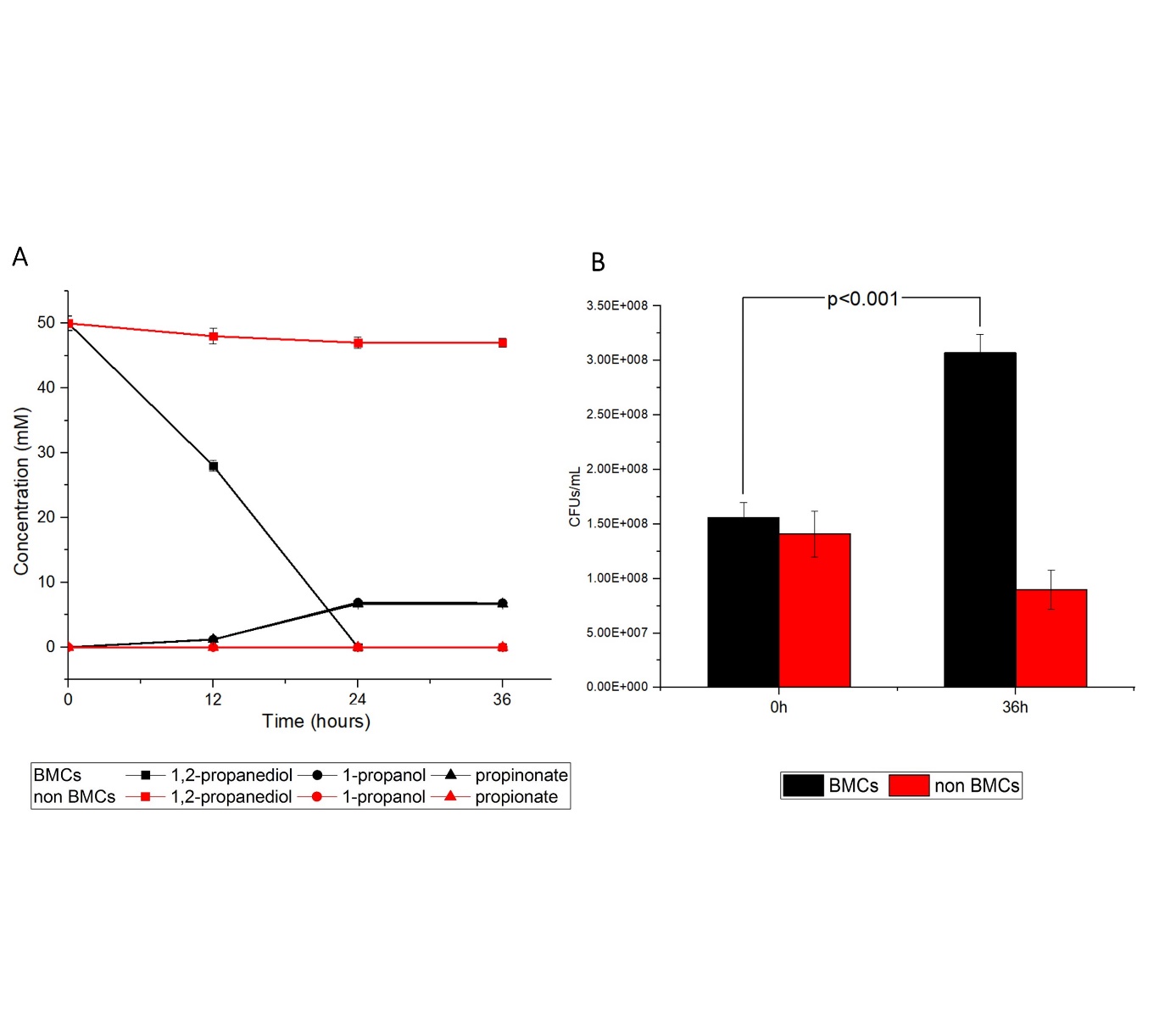


**SI Figure 4. 1,2-propanediol utilization and product formation (A) and impact on CFUs of *L. monocytogenes* EGDe (B) in MWB defined medium in anaerobic conditions**

1. Utilization of 1,2-propanediol (squares), production of 1-propanol (circles) and production of propionate (triangles); Pdu induced (in black) and Pdu control cells (in red). Results from three independent experiments with three technical repeats are expressed as mean ± s.e.m.
2. BMCs and non BMCs represent Pdu-induced cells with BMCs present, and non-induced control cells without BMCs, respectively; indicated p value from two-sided T-test.
